# Characterization and genomic analysis of *Salmonella* Abortusequi phage, vB_SalP_LDDK01, and its biocontrol application in donkey meat

**DOI:** 10.3389/fcimb.2024.1527201

**Published:** 2024-12-23

**Authors:** Shengliang Cao, Xinyu Kong, Yixuan Liu, Zhiwei Wang, Zhi Zhang, Xiaojing Lei, Pan Li, Liting Wang, Fan Yang, Shiyang Liu, Rongyue Li, Yubao Li, Xiujuan Feng, Tongtong Wang

**Affiliations:** ^1^ College of Agriculture and Biology, Liaocheng University, Liaocheng, Shandong, China; ^2^ School of Pharmacy and Food Engineering, Liaocheng University, Liaocheng, Shandong, China; ^3^ Police Dog Research Institute, NanJing Police University, Nanjing, Jiangsu, China

**Keywords:** *Salmonella* Abortusequi, phage vB_SalP_LDDK01, biological characterization, genomic analysis, biological control

## Abstract

*Salmonella* Abortusequi (*S.* Abortusequi) is the primary cause of abortions in equine animals, and can cause serious foodborne illness. Thus, effective biocontrol strategies are needed to decontaminate and control the emergence of foodborne diseases. In recent years, phages have been used as a new strategy for modulating foodborne pathogens and food safety. In this study, a new phage, vB_SalP_LDDK01, was isolated from donkey farm bedding. The data indicated that the incubation period of vB_SalP_LDDK01 was 10 min, the burst size was 378 PFU/cell, as well as a wide range of heat resistance (40-70°C) and pH stability (4-12). Furthermore, genomic analysis and electron microscopy indicated that vB_SalP_LDDK01 belongs to the Class *Caudoviricetes* and genus *Jerseyvirus*. Moreover, its genome was 42,378 bp long, encoded 57 ORFs, was double-stranded DNA with a 49.52% GC content, and lacked virulence and drug-resistant genes. In addition, how vB_SalP_LDDK01 inhibits the growth of *S.* Abortusequi and removes the biofilm of *S.* Abortusequi was assessed in a liquid broth medium, and the results showed that vB_SalP_LDDK01 inhibited the growth of *S.* Abortusequi for about 8 h and significantly reduced the viable bacteria abundance compared with the phage-free positive control. Further, vB_SalP_LDDK01 treated the host bacteria for 12 h and effectively destroyed the biofilm of *S.* Abortusequi. This study further investigated how effectively vB_SalP_LDDK01 modulates bacterial contamination in donkey meat inoculated with *S.* Abortusequi LCU-S-ABORT-F at 4°C and 25°C. Furthermore, after 72 h of vB_SalP_LDDK01 treatment with different multiplicity of infection (1, 0.1, 0.01, and 0.001), the bacterial contamination on the surface of donkey meat was reduced by 4.3, 3.7, 3.3, and 3.5 log_10_ CFU/piece at 25°C, and 4.5, 3.9, 2.8, and 2.7 log_10_ CFU/piece at 4°C. Whereas the phage titers at different temperatures were basically comparable to the initial titers. Overall, these results indicated that vB_SalP_LDDK01, the new phage, can serve as an effective biological agent and inhibit *S.* Abortusequi in donkey meat.

## Introduction

Salmonellosis, caused by *Salmonella*, is one of the most essential zoonoses (Zhu et al., 2021) and can cause diarrhea in humans, affecting one tenth of the world’s population (Wang et al., 2024b). *Salmonella* is frequently found as a foodborne pathogen and causes food poisoning, the most common disorder caused by bacterial poisoning (Wiedemann et al., 2015). Furthermore, it can infect various animals, such as mammals, birds, and humans (Grandolfo et al., 2018). Moreover, it has also been observed that *Salmonella* Abortusequi (*S.* Abortusequi) is the primary causative agent of equine abortion, which is characterized by mare abortion, neonatal septicemia, and polyarthritis (Bustos et al., 2016; Wang et al., 2019; Zhu et al., 2021). Since the 1980s, several studies have reported equine abortive salmonellosis worldwide; however, it has not been studied comprehensively (Wang et al., 2023). Over the past decade, high rates of equine abortion caused by *S.* Abortusequi infections have re-emerged in China, with 30% to 100% current abortion rates in infected mares (Wang et al., 2023), which results in significant economic losses to the Chinese equine economy.

China is one of the largest donkey-farming countries in the world (Seyiti and Kelimu, 2021). Because of its nutritional value and unique flavor, donkey meat is becoming increasingly popular, accounting for > 80% of the economic benefits of donkey farming and production (Li et al., 2020a; Man et al., 2023). In the last decade, a high incidence of *Salmonella abortus* has been reported in equids; therefore, the food safety of donkey meat is becoming a research hotspot. A study isolated a pathogen from a food poisoning diarrhea patient in Nanjing, China, which was significantly associated with *Salmonella* extracted from five-spice donkey meat (Du et al., 2018). In China, *Salmonella* infections in humans are primarily associated with foods of animal origin, such as dairy and meat products, and non-animal foods, including fresh fruits and vegetables (Wang et al., 2024b). The current measures to control bacterial contamination in the food include biological, physical, and chemical methods (Yeh et al., 2018; Rossi et al., 2023). However, these methods have adverse effects, such as food toxicity and quality alterations (Troy et al., 2016). For instance, it has been reported that chemical residues affect the flavor and structure of food (Huang et al., 2018), physical disinfection methods such as ultraviolet light causes the loss of food nutrients (Ravindran and Jaiswal, 2019), and excessive and unscientific use of antibiotics increases bacterial resistance and food safety problems (Beier et al., 2011; Wang et al., 2024a). Therefore, it is urgent to develop safe and effective methods to control *Salmonella* contamination in food.

Phages are bacteria-killing viruses that are specific, safe, have no dangerous effects on human or animal health, and are abundant in the environment. Therefore, phage methods are increasingly being applied to prevent and control bacterial contamination of food. Several studies have reported the use of phage preparations as an alternative to or in addition to antibiotic treatment to control the contamination of various food products with *Salmonella*, *Clostridium perfringens*, *Escherichia coli*, and other bacteria (Kutateladze and Adamia, 2010; Principi et al., 2019). *Salmonella* phage D1-2 has been observed to inhibit the growth of drug-resistant *Salmonella* in egg white and yolk liquid at 4°C and 25°C (Li et al., 2020b). Furthermore, at low temperatures, a cocktail of phages vB_SenM_P7 and vB_SenP_P32 in combination with propionic acid (PA) under modified atmosphere packaging (MAP) conditions can effectively control *Salmonella* in poultry meat, thereby improving food quality and safety (Pelyuntha and Vongkamjan, 2023). Moreover, *Vibrio parahaemolyticus*-specific phage vB_VpaS_OMN effectively inactivates *Vibrio parahaemolyticus* on the surface of oyster meat (Zhang et al., 2018). Furthermore, phage LPST10 has been found to effectively reduce *Salmonella* abundance in milk, sausages, and lettuce; thus, it is a promising candidate for controlling *Salmonella* contamination in food (Huang et al., 2018). A study revealed that phage vB_CpeP_HN02 inhibited 95 ~ 99% of *Clostridium perfringens* on the surface of chicken meat after 72 h of action at 4°C (Tian et al., 2022). Duc, H.M. et al. indicated that multivalent phage PS5 simultaneously controlled *Salmonella enteritidis*, *Salmonella typhimurium*, and *Escherichia coli* O157:H7 in foods such as chicken skin, raw beef, fresh lettuce, and milk (Duc et al., 2020).

In view of the serious harm of *S.* Abortusequi and the low number of studies on *S.* Abortusequi phage in recent years as well as the prevention and control of *S.* Abortusequi contamination in donkey meat, this study employed LCU-S-ABORT-F as the host bacterium to isolate phage vB_SalP_LDDK01 (hereafter referred to as LDDK01) from the bedding of a donkey farm in Liaocheng City, Shandong Province, China. The biological properties, morphology, and lysis ability of LDDK01 against the host bacteria in a liquid broth (LB) medium were assessed. Furthermore, the phage genome was sequenced and comprehensively analyzed. Moreover, the effect of LDDK01 on the growth of *S.* Abortusequi present on the surface of donkey meat was also investigated. The results revealed that LDDK01 can effectively inhibit the viable counts of *S.* Abortusequi and remove the biofilm of *S.* Abortusequi, thereby controlling its contamination in the food.

## Materials and methods

### Bacterial strain

All strains used in this study were obtained from the Phage Research Center of Liaocheng University, and the specific information is shown in **Table 1**. The methods for the isolation of avian *Salmonella* are described in previously published articles (Cao et al., 2022), whereas *S.* Abortusequi of donkey origin was isolated into the lungs, hearts, livers, and spleens of donkeys suspected of having *S.* Abortusequi in various donkey farms in Shandong Province. Briefly, the lung, heart, liver and other tissues of aborted donkey foals were aseptically taken and inoculated in LB liquid medium for bacterial enrichment, and then streaked and cultured on Nutrient Agar and Salmonella Shigella (SS) Agar, respectively, and a single suspected colony was picked for purification culture, and then the purified strains were subjected to polymerase chain (PCR) and serotype identification (Wang et al., 2023).

**Table 1:** Lytic activity of phage LDDK01 against *Salmonella*.

Strain	Type	Lytic activity	Cities ^a^	Separation date	Source
LCU-S-Abort-A	*S.* Abortusequi	+	Liaocheng	2021	Donkey
LCU-S-Abort-B	*S.* Abortusequi	+	Liaocheng	2021	Donkey
LCU-S-Abort-C	*S.* Abortusequi	+	Dezhou	2021	Donkey
LCU-S-Abort-D	*S.* Abortusequi	+	Dezhou	2021	Donkey
LCU-S-Abort-E	*S.* Abortusequi	+	Liaocheng	2022	Donkey
LCU-S-Abort-F	*S.* Abortusequi	+	Liaocheng	2022	Donkey
LCU-S-Abort-G	*S.* Abortusequi	+	Dezhou	2022	Donkey
S1	*Salmonella Typhimurium*	−	Weifang	2019	Chicken
S3	*Salmonella Enterica*	+	Liaocheng	2019	Chicken
S4	*Salmonella Enterica*	−	Liaocheng	2019	Chicken
S5	*Salmonella Enterica*	−	Liaocheng	2019	Chicken
S8	*Salmonella Enterica*	−	Dongying	2019	Chicken
S10	*Salmonella Typhimurium*	+	Yantai	2019	Chicken
S11	*Salmonella Typhimurium*	−	Yantai	2019	Chicken
S18	*Salmonella Pullorum*	−	Liaocheng	2020	Chicken
S19	*Salmonella Pullorum*	−	Liaocheng	2020	Chicken
S20	*Salmonella Pullorum*	−	Liaocheng	2020	Chicken
S22	*Salmonella Pullorum*	−	Liaocheng	2020	Chicken
S42	*Salmonella Enterica*	−	Liaocheng	2021	Duck
S44	*Salmonella Enterica*	−	Heze	2021	Duck
S104	*Salmonella Pullorum*	−	Zibo	2022	Chicken
S105	*Salmonella Pullorum*	−	Zibo	2022	Chicken
S224	*Salmonella Enterica*	+	Weifang	2023	Chicken
S278	*Salmonella Enterica*	+	Liaocheng	2023	Duck
S359	*Salmonella Enterica*	+	Liaocheng	2023	Duck
S527	*Salmonella Enterica*	+	Weifang	2023	Chicken

a Names of cities where the serum samples were located in Shandong, China

### Phage isolation, purification, and characterization

Phage was isolated from the bedding of aborted donkeys. Briefly, 200 μL of *S.* Abortusequi stored at -80°C is placed in a 10 mL centrifuge tube and then 5-7 mL of liquid LB medium is added and shaken for 12-16 h at 37°C at 180 rpm. The bacteria were then purified on SS medium by three-zone delineation method, and a single colony was picked and inoculated in LB liquid medium at 37°C with shaking at 180 rpm for 12 h. After incubation, the culture solution was placed in a 4°C refrigerator for further use. An appropriate amount of bedding material was sampled in a 50 mL centrifuge tube, mixed well with the appropriate amount of sterilized saline, and centrifuged at 10,000 rpm for 5 min at 4°C to collect the supernatant, which was then filtered through 0.22 µm filter membrane. Then 1 mL of the filtrate and 200 µL of bacterial solution were added in a 10 mL centrifuge tube, mixed with 5-7 mL of liquid LB medium for 4 h at 37°C and 180 rpm shaking. The culture was then centrifuged at 10,000 rpm for 5 min at 4°C, and the supernatant was filtered through a 0.22 µm filter membrane. Finally, the phage was verified by the dot-drop method, and the presence or absence of phage spots was observed (Lu et al., 2003).

Host bacteria (200 µL) was mixed well with 6-8 mL of LB semi-solid medium melted, cooled to 55°C, poured into the plate, mixed with 5 µL of filtrate dropwise and incubated at 37°C for 4-6 h. Then, the phage plaques were picked up in centrifuge tubes containing 1 mL of SM buffer, mixed well using vibration, and then centrifuged to filter. The double-layer plate method was employed to purify the phage in the filtrate. The above steps of phage plaques deduction, centrifugation, and filtration were repeated 3-5 times until phage plaques of uniform size and morphology were obtained.

### Phage potency assay

The double-layer plate method was used to prepare plates. Briefly, 10-15 mL of nutrient agar medium was melted and cooled to 55°C. The purified phage was diluted 10-fold, and the appropriate dilution was selected. Then, phage lysate (200 µL) and host bacteria (200 µL) were added in a 10 mL centrifuge tube, mixed well with 5-8 mL of LB semi-solid medium (melted and cooled to 55°C), and then poured into the configured plates placed in a 37°C incubator and cultured for 4-6 h. For each dilution, the experiments were repeated thrice.

### Phage host range

The host range of isolated and purified phage was assessed by spotting 19 avian *Salmonella* strains with different serotypes and 7 *S.* Abortusequi strains of donkey origin, respectively (Cao et al., 2022). The phage host range assay is mainly performed using the double-layer plate spotting method. Briefly, for plate preparation, 200 µL of bacterial solution was mixed with 5-8 mL of melted LB semi-solid medium cooled to 55°C. To observe the presence or absence of phage plaques, 10 µL of phage lysate was dropwise added to the plates and incubated at 37°C for 4-6 h. The presence of phage plaques indicated that the phage could lyse the strain. The experiment was repeated three times to obtain reliable results.

### Phage morphology analysis

For this analysis, the previously described method was followed (Cao et al., 2022). The phage was concentrated by double plate amplification to obtain highly efficient phage lysates for easy morphological analysis using a microscope. The phage lysate was negatively stained with 1% phosphotungstic acid, and the phage filtrate was added dropwise onto a copper mesh grid and then the morphology of the phage was observed using a JEM-1200EXII Transmission Electron Microscope (TEM) (JEOL, Japan) by imaging the isolated phage at an acceleration of 80 KV. The phage morphology was analyzed and measured using ImageJ software (Li et al., 2020b).

### Phage multiplicity of infection analysis

Using the 10-fold dilution method, the phage was diluted, and the appropriate dilution was mixed with host bacteria in a 1:1 ratio (500 µL each) at a concentration of 10^8^ CFU/mL at log_10_ growth phase (1, 0.1, 0.01 and 0.001 respectively) for 3 h at 37°C with 180 rpm shaking. Then, the phage potency was determined using a double-layer agar plate method, and the optimal MOI with the highest potency was selected. For each group, 3 parallel experiments were conducted.

### Phage one-step growth curve analysis

A one-step growth curve assay was performed with slight modifications of the previously described method (Sun et al., 2012) to assess the incubation period and phage outbreak size. The phage was diluted by the 10-fold dilution method, and the appropriate dilution was mixed with host bacteria in a 1:1 ratio (500 µL each) at a concentration of 10^8^ CFU/mL at log_10_ growth phase and incubated in a water bath at 37°C for 10 min. This mixture was then centrifuged at 10,000 rpm for 5 min at 4°C to remove the supernatant, washed twice with equal volume of 37°C pre-warmed LB medium, resuspended with 1 mL of LB medium, added with 10 mL of LB medium, and incubated at 37°C with constant shaking at 180 rpm. The mixture was sampled (500 µL) at 0 min and then once every 10 min. Samples were taken at intervals of 120 min, and phage titers were determined immediately in the first portion, and in the other portion after treatment with 1% chloroform for 30 min, and then phage titers were determined, and the test was repeated thrice for each time point. The steps for the determination of phage titer were as follows (Zhao et al., 2024). The samples were centrifuged at 10,000 rpm for 3 min, filtered through a 0.22 µm filter membrane to obtain the phage lysate, and then diluted with a 10-fold specific dilution method. The titer of phage in the lysate was determined using the double-layer plate method. The one-step growth curve of phage was plotted with incubation time on the horizontal axis and the phage potency logarithmic value on the vertical axis. Phage burst size was evaluated by dividing the average number of final free phage particles by the number of initial free phage particles (Shang et al., 2021).

### Phage thermal stability analysis

A total of 5 tubes of phage lysate (500 µL/tube) were incubated in a water bath at 40°C, 50°C, 60°C, 70°C, and 80°C, respectively. At each temperature, 100 µL of phage lysate was removed from each tube at 20, 40, and 60 min, respectively. The potency was assessed *via* the double-layer plate method. The experiment was repeated thrice.

### Phage pH stability analysis

Phage lysate (100 µL) was mixed with SM buffer (900 µL) at pH 1, 2, 3, 4, 5, 6, 7, 8, 9, 10, 11, 12, and 13, respectively, in a water bath at 37°C for 1 h. The potency of the lysate was determined by the double-layer plate method. The experiment was repeated thrice.

### Phage bioinformatics analysis

For phage DNA extraction, E.Z.N.A^®^ DNA kit (OMEGA, United States) was employed, whereas TruSeq™ Nano DNA Sample Prep Kit (Illumina, United States) was used for sequencing libraries. Genome sequencing was performed by Shanghai Lingen Biotechnology (Shanghai, China) using the Illumina novaseq6000 sequencing platform. Quality control raw data were filtered using fastp 0.23.4 (Chen et al., 2018), and FastQC 0.12.0 (Brown et al., 2017), and the genome was assembled using SPAdes 3.15.5 (Bankevich et al., 2012).

The open reading frames of phage were predicted using GeneMarkS 4.28 (Besemer et al., 2001) (http://topaz.gatech.edu/GeneMark/genemarks.cgi). Furthermore, the function of each ORF-encoded protein was predicted *via* the BLASTp (https://blast.ncbi.nlm.nih.gov/Blast.cgi?PAGE=Proteins ). Moreover, VFDB (Liu et al., 2019a) (http://www.mgc.ac.cn/cgi-bin/VFs/v5/main.cgi), CARD (Alcock et al., 2023) (https://card.mcmaster.ca/), ICEfinder (Liu et al., 2019b) (https://bioinfo-mml.sjtu.edu.cn/) were used, respectively, to predict the function of each ORF. Moreover, ICEfinder/ICEfinder.html predicted the genomic virulence genes, drug-resistance genes, and integrases. The ANI between genomes was calculated using JSpeciesWS (http://jspecies.ribohost.com/jspeciesws) (Richter et al., 2016) based on the Blast+ algorithm. The TBtools software was employed to plot ANI maps with ANI values. Phage LDDK01 has been uploaded to NCBI’s Genbank database under accession number PP932687.

### Biofilm removal ability of phage

The ability of phage LDDK01 to remove the host bacterial biofilm was determined by following the previous literature (Runci et al., 2017). Briefly, host bacterial solution (10^8^ CFU/mL, 200 µL) was added to a 96-well plate for 48 h at 37°C. Then, the culture solution was gently aspirated, washed with PBS buffer thrice, and incubated with phage LDDK01 lysate (200 µL) at 1, 0.1, 0.01, and 0.001 MOI. The control group received an equal volume of LB. The plates were incubated in a constant temperature incubator for 6 h and 12 h. Then, the wells were washed with 200 µL of PBS buffer, stained with 200 µL of 1% crystal violet solution at room temperature for 30 min, washed to remove the crystal violet solution thrice with sterile PBS buffer, and treated with 200 µL of 100% ethanol solution for 15 min to completely dissolved the biofilm. Lastly, 100 µL of this solution was sampled to measure the optical density (OD) at 595 nm with an enzyme meter.

### Lysis ability of phage

The *in vitro* lysis ability of LDDK01 was determined using the reference already published in the literature (Lu et al., 2003). Briefly, different concentrations of phage (MOI = 1, 0.1, 0.01, and 0.001) were mixed with the host bacterial solution (10^8^ CFU/mL), respectively. The positive control group was the host bacterial culture medium without phage, while the negative control group included phage and LB medium; both the groups were incubated at 37°C for 24 h. The culture solution of each group was collected at 2-h intervals to measure the OD of each group at 600 nm. Each experiment was repeated three times.

### Inhibition of *S.* Abortusequi in donkey meat by phage LDDK01

The bacteriostatic effect of LDDK01 on *S.* Abortusequi present on the surface of donkey meat was verified based on the relevant methods described in the literature (Shang et al., 2021; Noor Mohammadi et al., 2022; Tian et al., 2022). Freshly slaughtered donkey meat was purchased from the slaughterhouse in Liaocheng City, Shandong Province. To reduce contamination, the meat (approximately 0.5 g) was sliced into 1 × 1 cm squares in a sterile biosafety cabinet, placed in a sterile culture dish, and disinfected with ultraviolet radiation. During the sterilization process, the petri dish was turned over twice, etc. After sterilization, donkey meat from different areas was randomly taken for bacterial isolation and identification, and phage LDDK01 biocontrol experiments were carried out after detection of sterility. Briefly, 50 μL of *S.* Abortusequi (10^8^ CFU/mL) was added to 5 spots (10 μL/spot) on the surface of each sterile donkey meat and incubated for 1 h in a sterile biosafety cabinet. Then, 100 μL of LDDK01 at 1, 0.1, 0.01, and 0.001 MOI were taken, added to the same location, and immediately placed in sterile sealed bags at 4°C and 25°C for 0, 1, 12, 24, 36, 48, and 72 h, respectively. For the positive control group, the same volume of sterile PBS was used instead of phages. At each sampling time point, donkey meat pieces were placed in 2 mL of sterile PBS buffer, minced with a tissue homogenizer, and then evenly divided into two parts. One part was centrifuged and diluted with PBS gradient for bacterial counting using SS medium at 37°C. The other part was centrifuged and filtered through a membrane to obtain a phage solution. The phage potency was determined at 37°C using a double-layer plate method. Each experiment was repeated thrice.

### Statistical analysis

Bacterial counts and phage titers were determined by plate counting and double-layer plate methods, respectively. Furthermore, bacterial counts were converted to log_10_ CFU/mL or log_10_ CFU/piece, while the phage counts were converted to log_10_ PFU/mL. All statistical analyses were performed using the SPSS 23.0 software (SPSS Inc., version 27.0; Chicago, IL, USA), and the data are expressed as the mean ± standard deviation (SD) of at least three biological replicates. Statistical differences between groups were assessed using Student’s t-test. *p-*values < 0.05 indicated a statistically significant difference (^ns^ *p* > 0.05, * *p* < 0.05, ** *p* < 0.01, and *** *p* < 0.001).

## Results

### Isolation, purification, and morphology of phage LDDK01

**Figure 1:**
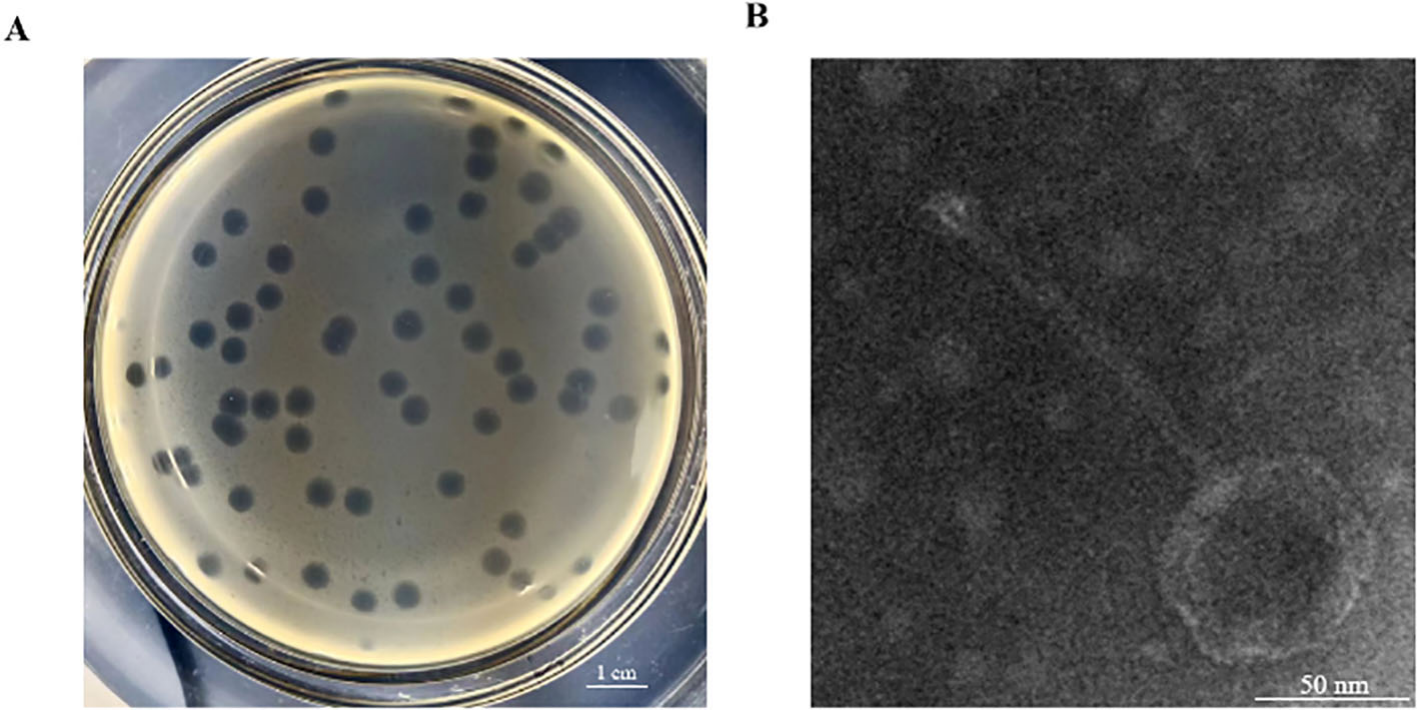
Isolation, purification, and morphology of LDDK01. **(A)** Phage plaques formed on double-layer agar plates. **(B)** Transmission electron micrograph of phage LDDK01. Scale bar = 50 nm.

This study isolated an *S.* Abortusequi phage, named vB_SalP_LDDK01, from the bedding of a donkey farm in Liaocheng City, Shandong Province, China. The phage was purified using the double-layer plate method and then used to verify the host spectrum of 7 *S.* Abortusequi strains and 19 avian *Salmonella* strains with different serotypes (**Table 1**). The results showed that phage LDDK01 lysed the 7 strains of *S.* Abortusequi, 2 strains of *Salmonella typhimurium*, and 4 strains of *Salmonella enteritidis* (**Table 1**; **Supplementary Figure S1**). Furthermore, it formed transparent, regular, and uniformly sized round phage plaques on double-layer plates (**Figure 1A**). LDDK01 is a phage that has not been reported by other researchers. Therefore, it was further investigated. The TEM analysis of the purified LDDK01 revealed that it had an icosahedral head with a diameter of 54.8 nm and a long, non-contractile tail with a diameter of 120.6 nm (**Figure 1B**), suggesting that the phage belongs to the *Siphoviridae* family.

### Biological characterization of phage LDDK01

**Figure 2:**
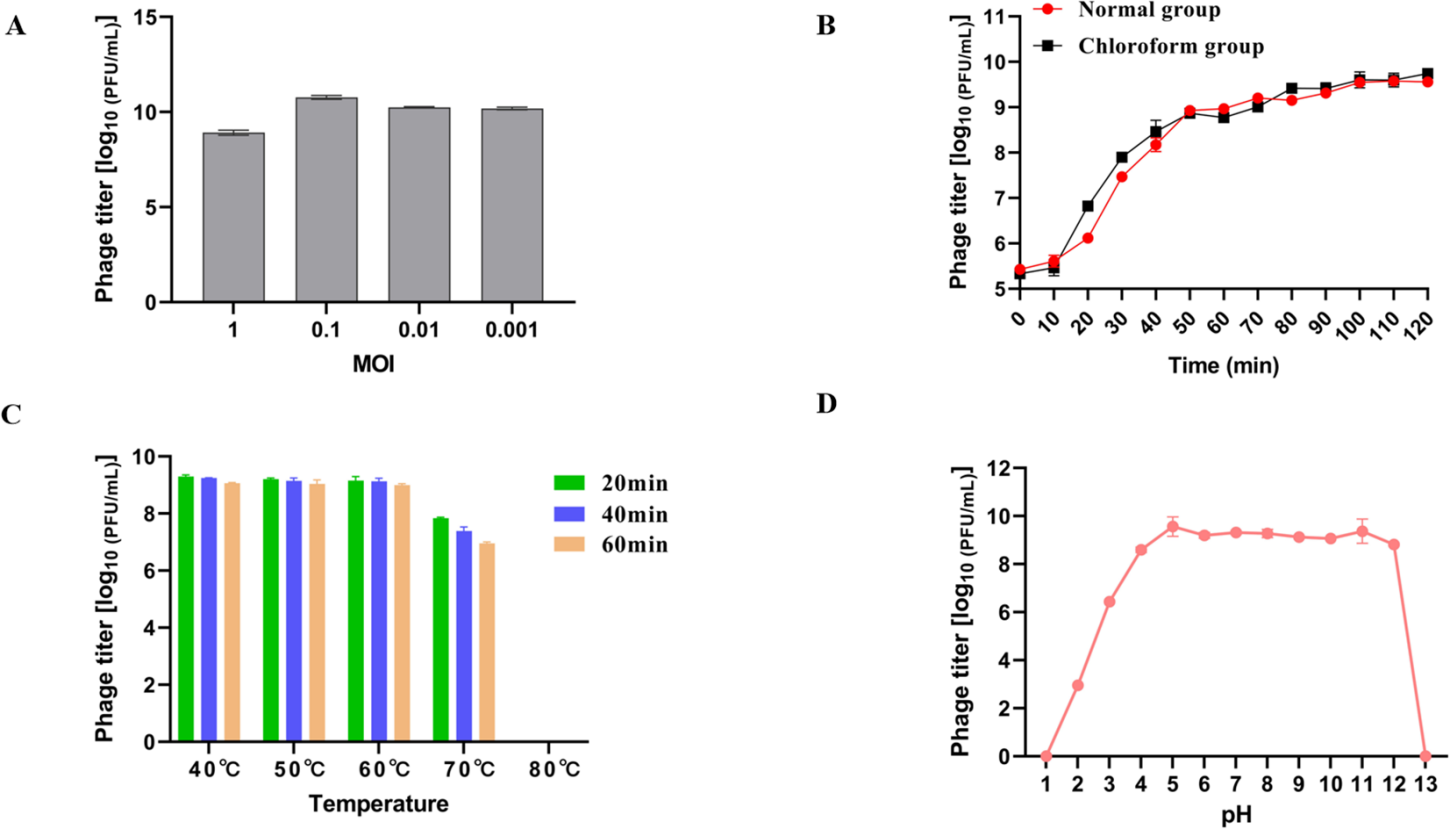
Biological characterization of phage LDDK01. **(A)** Optimal multiplicity of infection. **(B)** One-step growth curve of phage LDDKO1 in host strain *S.* Abortusequi LCU-S-ABORT-F. **(C)** Stability of phage at different temperatures ranging between 40-80°C. **(D)** Stability at different pH ranging between 1 and 13. The phage titers were determined using the double-layer agar method, and each data point represented the mean values ± standard deviations (SD) of at least three replicate experiments.

Different concentrations of LDDK01 and host bacteria were mixed and incubated for 3 h to determine the phage titers by the double-layer plate method (**Figure 2A**). It was observed that the phage potency was the highest at the MOI of 0.1, which was about 1.86 × 10^10^ PFU/mL; therefore, the optimal MOI of this phage was 0.1 (**Figure 2A**). One-step growth curve analysis showed that the incubation period of the phage was about 10 min. Furthermore, after the phage infected the host bacteria, its potency increased rapidly in 10-50 min and stabilized after 60 min (**Figure 2B**). The burst size of LDDK01 was calculated to be about 378 PFU/cell.

The heat resistance analysis revealed a stable phage potency at 40, 50, and 60°C for 1 h. After incubation at 70°C for 20 min, 40 min, and 1 h, the phage potency decreased by about 1.2 log, 1.7 log, and 2 log_10_ PFU/mL, respectively. Moreover, its activity was completely lost at 80°C after only 20 min (**Figure 2C**). Similarly, LDDK01 maintained a high potency at pH 4-12; however, its potency was significantly reduced from pH 2 and 3 to 3 and 6.4 log_1010_ PFU/mL, respectively. At pH 1 and 13, the phage is completely inactivated under strong acid and alkali conditions (**Figure 2D**).

### Bioinformatics analysis of phage LDDK01

**Figure 3:**
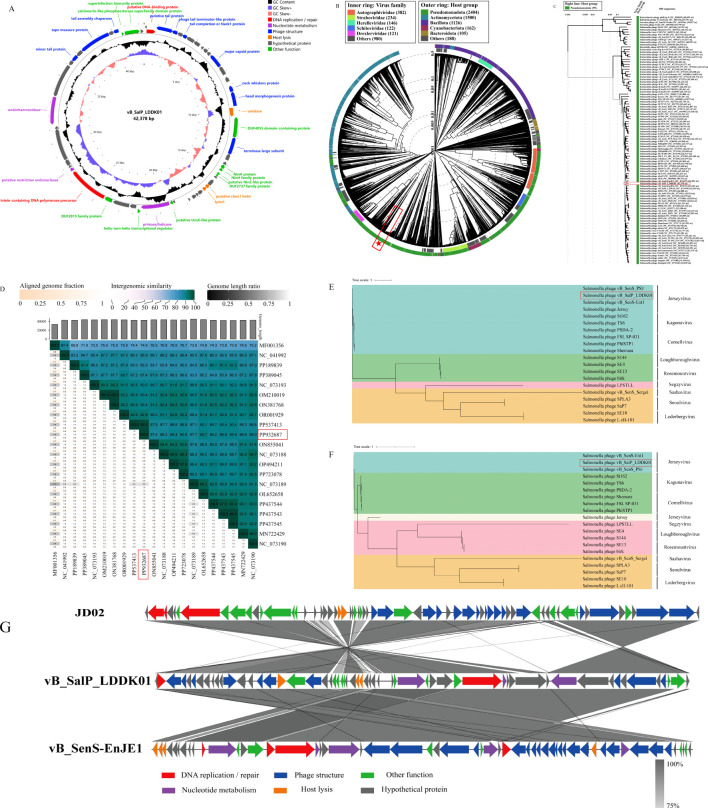
Comparative genomics analysis of *S.* Abortusequi phage LDDK01. **(A)** Circle diagram of phage LDDK01 genome. Meaning of the circle diagram from outside to inside: CDS with unused functions are indicated by different colors, CDS in the negative strand is in the outermost ring, CDS in the positive strand is in the inner ring, GC content higher than the average content of the genome is the outer peak, GC content lower than the average content is the inner peak, GC content offset (blue represents the > 0 offset value and the orange-red color indicates < 0 offset value), genome size. **(B)** Phage LDDK01 formed a circular proteomics tree with 5633 prokaryotic dsDNA viruses. The position of LDDK01 is marked with a red “pentagram”. **(C)** LDDK01 also formed a phage rectangular proteomics tree similar to the proximity in Figure **(B)**. The position of LDDK01 is marked with a red line. **(D)** Heatmap of intergenomic similarity between phage LDDK01 and its related phages. The right half indicates the similarity values between phage genomes, and the left indicates aligned genome scores and length ratios. The position of LDDK01 is marked with a red line. **(E)** Phage LDDK01 phylogenetic evolutionary trees based on major coat proteins and **(F)** the large subunit of the terminal enzyme. **(G)** Covariance analysis of the LDDK01 genome. Different colored arrows indicate the predicted ORFs and transcriptional directions. The color intensity from light gray to gray represents the amino acid sequence similarity (75-100%).

The whole genome sequence analysis revealed that LDDK01 had a 42,378 bp long genome with double-stranded DNA, 49.52% GC content, and encoded 57 ORFs. Furthermore, the annotation results showed that the ORFs of LDDK01 had 28 known proteins and 29 hypothesized proteins. The phage lacked tRNAs and did not carry virulence and drug-resistance genes. The predicted functional proteins primarily included DNA replication and modification modules (DNA ligase, endonuclease, *etc*.) and phage structural modules (tail protein, major coat protein, *etc*.). The annotation results of known functions of the phage genome are shown in **Supplementary Table S1**, and the genome circle diagram is shown in **Figure 3A**.

For the isolated phage LDDK01, a proteomic tree was established using 5633 phage genomes acquired from the Viptree database. It was observed that LDDK01 and vB_SenS-EnJE1 were clustered together with a close affinity, and their bacterial hosts all belonged to the class Pseudomonadota (**Figures 3B, C**). The BLASTn comparison of the NCBI database revealed that the highest sequence homology (99.94%) was between the genome of LDDK01 and *Salmonella* phage vB_SenS_4FS1 (PP537413.1), followed by *Salmonella* phage PIZ SAE- 01E2 (MN336266.1) with 94.78% homology. The similar phage genome sequences were extracted and further analyzed using VIRIDIC (**Figure 3D**), which revealed that LDDK01 indicated the highest homology with *Salmonella* phage JD02 (OL652658), followed by *Salmonella* phage vB_SenS_Psm105 (PP437544) and *Salmonella* phage vB_SenS_Psm140 (PP437543).

The phylogenetic relationships between LDDK01 and 19 *Salmonella* phages were assessed based on two representative highly conserved proteins: the major coat protein (LDDK01-ORF12) and the terminase large subunit (LDDK01-ORF19). LDDK01 indicated a significant association with three phages of the genus *Jerseyvirus*, located in the same branch and distantly related to phages of eight other genera (**Figure 3E**). Furthermore, a similar evolutionary tree was obtained when the amino acid sequence of the terminase large subunit was employed (**Figure 3F**), which indicated a close association with two strains of *Jerseyvirus* and a distant affinity to *Salmonella* phage Jersey. Moreover, the genome sequences of LDDK01 were compared with those of the homologous phages vB_SenS-EnJE1 and JD02, respectively, using Easyfig to analyze the genome evolutionary relationships (**Figure 3G**). The data indicated that LDDK01 was significantly correlated with vB_SenS-EnJE1, showing similarities in the protein’s function and the regions where they were located. LDDK01 and JD02 showed opposite regional similarities. Overall, based on the morphology assessed *via* electron microscopy, comparative genomic analysis, phylogenetic analysis, and the latest guidelines of the International Committee on Classification of Viruses (ICTV), LDDK01 was determined as a new phage belonging to the Class *Caudoviricetes* and genus *Jerseyvirus.*

### *In vitro* bacteriostatic effect and biofilm elimination ability of phage LDDK01

**Figure 4:**
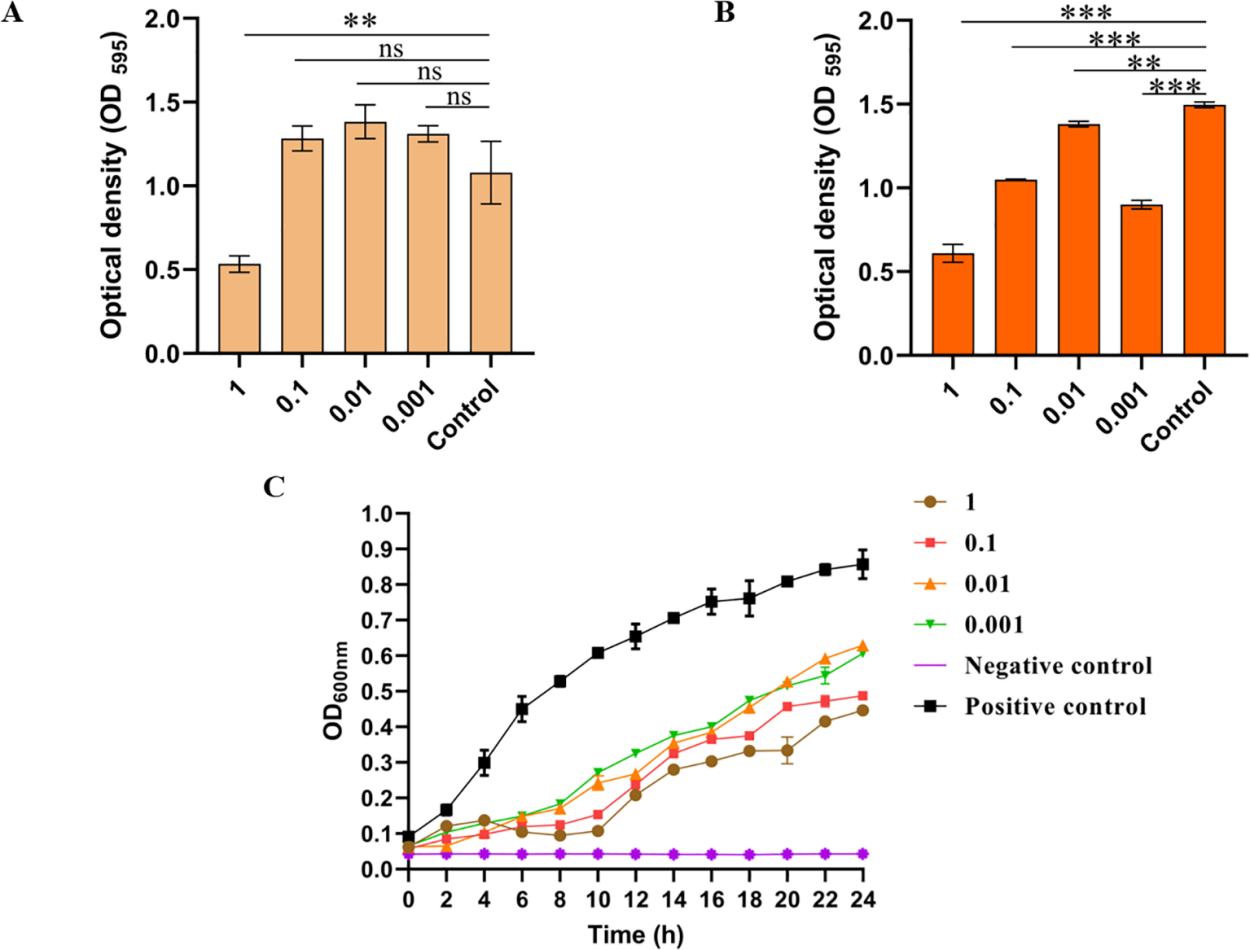
*In vitro* bacteriostatic effect and biofilm elimination ability of phage LDDK01. **(A)** The ability of phage LDDK01 to remove biofilm when treated for 6 h and **(B)** 12 h. ^ns^ *p* > 0.05, *p < 0.05, **p < 0.01, ***p < 0.001 compared to the control group sample. The control group had the same volume of LB medium. **(C)** *In vitro* inhibition curve of phage LDDK01. The positive control group included a host bacterial culture medium without phage, while the negative control group comprised the phage and LB medium. Error bars show the standard deviation of the mean (n = 3).

To verify the ability of LDDK01 to remove the biofilm of *S.* Abortusequi, the bacterial biofilm was treated with phage treatment and then subjected to the biofilm crystallization violet method. The results showed that only at MOI = 1, 6 h of phage treatment could remove the biofilm (**Figure 4A**). At 12 h of phage treatment, the phage significantly removed the biofilm (**Figure 4B**). These results suggest that LDDK01 can remove the *S.* Abortusequi biofilm. Furthermore, the *in vitro* inhibition of host bacteria within 24 h after LDDK01 treatment at different MOIs indicated that LDDK01 had good inhibitory effects in different infection complex groups compared with the bacterial fluid-positive control group. Moreover, the inhibitory effect of LDDK01 was similar at different MOI groups from 0 to 8 h, where the inhibitory effect at MOI = 1 was better than at other MOI after 8 h (**Figure 4C**).

### Effectiveness of phage LDDK01 in reducing *S.* Abortusequi in donkey meat

**Figure 5:**
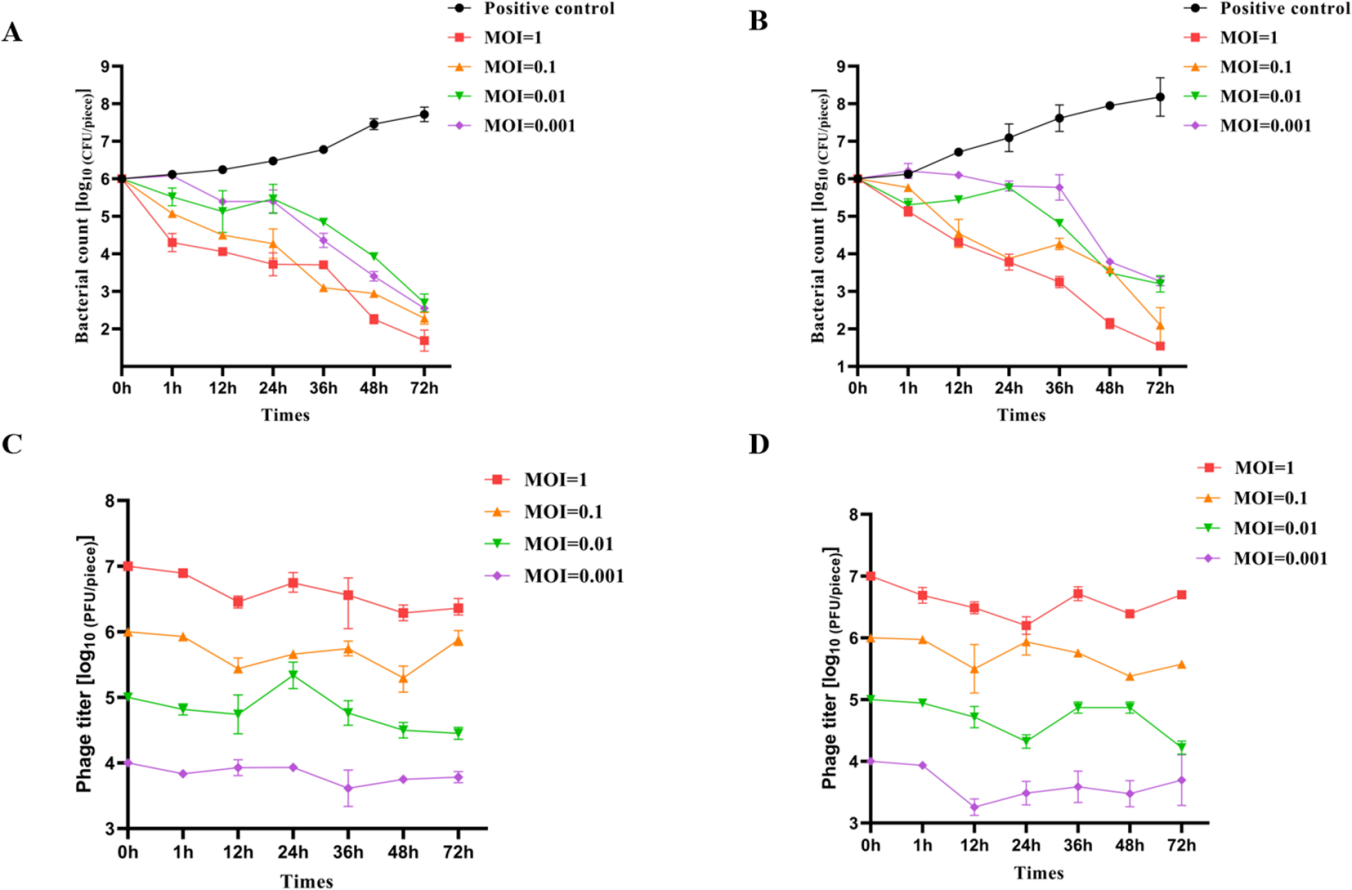
Effectiveness of phage LDDK01 in reducing *S.* Abortusequi in donkey meat. **(A)** Levels of *S.* Abortusequi in donkey meat at 25°C and **(B)** at 4°C. **(C)** The potency of *S.* Abortusequi phage in donkey meat at 25°C and **(D)** at 4°C. Error bars show the standard deviation of the mean (n = 3).

This study investigated the inhibitory effect of LDDK01 on *S.* Abortusequi present on the surface of donkey meat. The sterile donkey meat pieces were artificially infected with *S.* Abortusequi and then treated with LDDK01 with different MOIs for different times at 25°C and 4°C, respectively. The results showed that LDDK01 significantly inhibited *S.* Abortusequi in donkey meat at different temperatures, and the bacterial inhibition rate was essentially proportional to the phage’s titer. The bacterial content of each group 4.3, 3.7, 3.3, and 3.5 log_10_ CFU/piece after 72 h of LDDK01 treatment at different MOIs (1, 0.1, 0.01, 0.001) was compared with that of levels of *S.* Abortusequi in donkey meat from initial inoculation at 25°C (**Figure 5A**). Compared with the levels of *S.* Abortusequi in donkey meat from initial inoculation at 4°C, the bacterial content of each group decreased by 4.5, 3.9, 2.8, and 2.7 log_10_ CFU/piece, respectively, after 72 h of LDDK01 treatment at different MOI (**Figure 5B**). These data suggested that LDDK01 is more effective in controlling *Salmonella* populations at 4°C than at 25°C. The phage assay (**Figures 5C, D**) showed that during the inhibition process at 25°C and 4°C, each group’s phage titers did not change significantly and were comparable to the initial titers.

## Discussion

*S.* Abortusequi is the primary pathogen responsible for abortion in equine animals, which is frequently spreading in various regions. Due to the prolonged pregnancy of equine animals and the small number of offspring in each litter, abortion causes great economic losses to the donkey industry in China (Wang et al., 2023). Recently, the food safety of donkey meat has gained increasing attention because of the high prevalence of *S.* Abortusequi in equine animals (Wang et al., 2019). With the growing demand for alternatives to antibiotics and chemical sanitizers (Beier et al., 2011; Troy et al., 2016), phages have gained much attention for their application as alternative biocontrol agents for controlling pathogenic bacterial contamination in food, food preparation, and food processing environments. This study isolated and characterized an equine *S.* Abortusequi phage, LDDK01, from the bedding of a donkey farm in Liaocheng, Shandong Province, China. LDDK01 is a new phage that can infect equine *S.* Abortusequi and some avian *Salmonella* and can be used as a biocontrol agent in the food industry.

The stability of phage is essential for the effectiveness and shelf life of food biocontrol. Temperature and pH tolerance are the key factors that can significantly affect phage activity. Therefore, this study evaluated the phage’s ability to tolerate different temperatures and pH values to assess its clinical potential and efficiency. Wang et al. isolated phage P_IZ_ SAE-01E2 from sewage and submitted it to temperatures ranging from 4 to 70°C and pH values between 4 and 10 for 80 min (Wang et al., 2020). The data revealed that the abundance of phage remained high. Furthermore, Yuqing Zhou et al (Zhou et al., 2022). isolated phage EP01, which indicated stable potency when subjected to 40-60°C temperature for 1 h; however, the potency decreased by 4 log_10_ units after 1 h at 70°C. The change in potency was small at pH 5-10; however, at pH 2, 3, 12, and 13, the phage became inactivated. Here, the isolated phage LDDK01 indicated good heat, acid, and alkali resistance and can maintain good activity at 40-70°C and pH 4-12. After 1 h of LDDK01 treatment at pH 2-4, the potency decreased significantly; however, there was still a certain degree of activity. These data indicate that LDDK01 can be used in different pH and temperature food matrices.

Phage MOI is an important bioindicator that measures the amount of phage inputs and outputs. A smaller MOI can reduce the cost of phage production, which is helpful for the large-scale production and application of phage products (Abedon, 2016). Here, the optimal MOI of LDDK01 was 0.1, and its potency was up to 1.86 × 10^10^ PFU/mL, indicating that this phage can achieve the best bactericidal effect in the clinical setting. During phage adsorption with host bacteria, the replication efficiency of phage is enhanced by the short latency period, fast lysis rate, and large lysis volume, allowing the rapid generation of progeny viruses. Yating Guo et al (Guo et al., 2021). isolated the *Salmonella* phage LPSTLL and revealed that it had an incubation period of 10 min, a lysis period of 120 min, and a lysis volume of about 71 PFU/cells. Furthermore, Yuqing Zhou et al (Zhou et al., 2022). isolated the *Escherichia* phage EP01 from swine farm effluent, which had an incubation period of about 10 min and a lysis volume of about 80 PFU/cell. Here, the one-step growth curve showed that the latency period of phage LDDK01 was about 10 min. Moreover, its potency increased in the first 50 min but stabilized after 60 min, and the final phage potency was maintained at 10^9^-10^10^ PFU/mL. Therefore, in this study, the isolated and purified phage has the advantages of short latency and fast lysis, further confirming their potential as food biocontrol agents.

Bacterial biofilm is an excessive bacterial aggregated membrane-like material formed when bacteria attach to contact surfaces, secrete polysaccharide substrates as well as fibrous and lipid proteins, and wrap themselves around them (Baldassarri et al., 2001). Antibiotics do not affect bacteria in the biofilms, making it challenging to treat infections (Baldassarri et al., 2001). In this study, LDDK01 significantly degraded the bacterial biofilm after 12 h of treatment, and this ability had the same efficiency as phage EFDG1 (Khalifa et al., 2015), further demonstrating the potential of this phage to replace antibiotics. However, when phage LDDK01 treated its host bacteria for 6 h, only phage with MOI of 1 was able to inhibit the formation of bacterial biofilm, whereas phages with different MOIs were able to inhibit the formation of bacterial biofilm when the host bacteria were treated for 12 h. These results may indicate that the ability of phage LDDK01 to remove bacterial biofilm may be related to the amount of phage and the duration of action.

Whole genome sequencing is recognized as an essential tool to ensure the safety of phage preparation (Shang et al., 2021) and understand phage characteristics (Jin et al., 2023). Phages contain virulence and drug-resistance genes that limit their application in food biocontrol in the food industry. Whole genome sequencing was carried out to assess the genetic characterization of phage LDDK01, which showed that LDDK01 lacks virulence and drug-resistance genes, indicating its suitability as an alternative for the food hygiene industry. Furthermore, the whole-genome sequence and phylogenetic analysis of the telomerase large subunit and major coat protein of phage LDDK01, as well as gene annotation, revealed that LDDK01 is a new phage belonging to the genus *Jerseyvirus* of Class *Caudoviricetes*. Further classification of this phage is warranted to better understand its biodiversity.

One of the main criteria for selecting phage for developing biocontrol against foodborne pathogens is the phage’s lysing ability and stability (Montso et al., 2019). In this study, donkey meat was artificially inoculated with *S.* Abortusequi and then phage-treated at 4°C and 25°C to simulate industrial storage and processing temperatures. LDDK01 treatment was given at different temperatures for 72 h, significantly reduced the viable counts of *S.* Abortusequi in donkey meat (average 3.6 log_10_ CFU/piece) compared with its initial inoculated levels, indicating that the significant bacterial inhibitory effect of LDDK01, even in a refrigerated environment. Several studies have reported that phages can reduce *Salmonella* contamination in food, such as LPST94 (Islam et al., 2020), S144 (Gambino et al., 2020), *etc.* Tahir et al (Noor Mohammadi et al., 2022). treated *Clostridium perfringens* with phage CPQ1 at different temperatures and observed that the viable counts of *Clostridium perfringens* increased in chicken even at the MOI of 10, suggesting that drug-resistant bacteria may regenerate, inconsistent with the presented study results. Here, 12 h LDDK01 treatment of *S.* Abortusequi in donkey meat at different temperatures resulted in decreased (by 1 log_10_ CFU/piece on average) viable bacteria abundance in donkey meat, which further decreased by 3.6 log_10_ CFU/piece on average after 72 h of treatment. The short incubation period and strong lytic ability of LDDK01 might indicate why surface-resistant bacteria are not easily produced in donkey meat.

In general, the higher the MOI of the phage in the food, the higher the pathogenic bacteria reduction because the high phage titer increases the possibility of phage binding to the host bacteria. This study also revealed that the high titer of LDDK01 had a significant bacteriostatic effect in donkey meat compared to its low titer. This is consistent with the study of Zhang et al., which showed that high doses of EP01 better removed bacterial contamination from food surfaces (Zhou et al., 2022). This may be because the high EP01 dose causes more collisions between the phage particles and the bacterial cells on the food surface (Zhou et al., 2022). Although phages cannot eliminate bacteria, they significantly reduce the number of viable bacteria, thereby decreasing the probability that consumers would be exposed to infectious doses of pathogenic bacteria.

Although LDDK01 can effectively remove the pathogen contamination from the surface of donkey meat, there are still certain limitations: (i) When applied on food products, LDDK01 can only target some bacteria and has a narrow host range, (ii) LDDK01 can only reduce the number of viable bacteria in the host below the initial level; however, cannot eliminate all the bacteria from the surface of the donkey meat, and (iii) The LDDK01 cross-species lysing ability against some avian *Salmonella* was not determined. Based on the above limitations, our future research will focus on combining LDDK01 with other phages or natural bactericidal products to target more bacterial species, prevent the emergence of drug-resistant strains, and achieve complete sterilization. Furthermore, the mechanism of LDDK01’s action on the host will be studied and the range of the hosts of phage LDDK01 will be expanded through genetic engineering (Shang et al., 2021).

## Conclusion

This study isolated a new phage strain, LDDK01, from the bedding of a donkey farm in Liaocheng City, Shandong Province, China. The biological characterization revealed that LDDK01 was safe and had high lysing ability, wide pH and thermal stability, a short incubation period, and high reproductive activity. Furthermore, the genomic DNA sequencing analysis revealed that LDDK01 lacked drug-resistant and virulence genes. Moreover, LDDK01 was able to eliminate bacterial biofilm and reduce the growth of *S.* Abortusequi in both LB medium and donkey meat. These data suggest that the phage LDDK01 can control foodborne pathogens and, therefore, be used as a natural alternative biocontrol agent for food production and preservation.

## Data Availability

The datasets presented in this study can be found in online repositories. The raw data from the sequence experiment have been deposited in the database of NCBI under accession numbers PP932687. (https://www.ncbi.nlm.nih.gov/).
