# Supplementary material for: Characterization and genomic analysis of Salmonella Abortusequi phage, vB_SalP_LDDK01, and its biocontrol application in donkey meat
Source: Front Cell Infect Microbiol. 2024 Dec 23;14:1527201. doi: 10.3389/fcimb.2024.1527201 (PMC11700823; doi:10.3389/fcimb.2024.1527201)
Supplement: Supplementary file 1 [file SupplementaryFile1.docx]

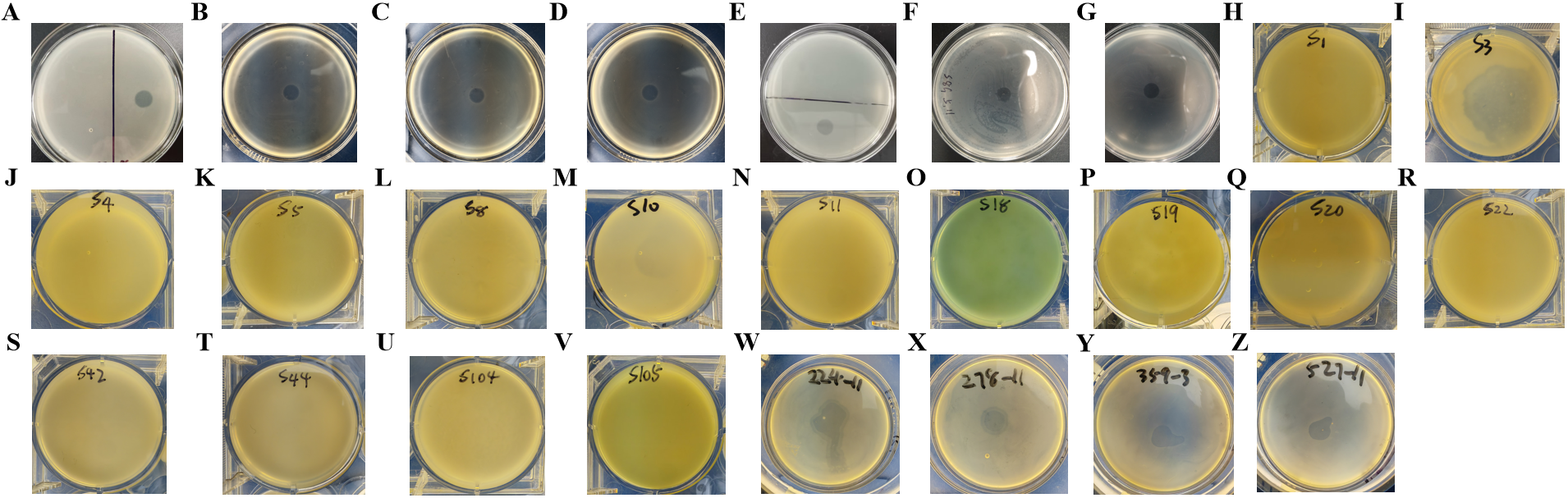


**Supplementary Figure S1. Validation of the host profile of phage LDDK01.**A) *S. Abortusequi* LCU-S-Abort-A. (B) *S. Abortusequi* LCU-S-Abort-B. (C) *S. Abortusequi* LCU-S-Abort-C. (D) *S. Abortusequi* LCU-S-Abort-D. (E) *S. Abortusequi* LCU-S-Abort-E. (F) *S. Abortusequi* LCU-S-Abort-F. (G) *S. Abortusequi* LCU-S-Abort-G.. (H) Avian *Salmonella* S1. (I) Avian *Salmonella* S3. (J) Avian *Salmonella* S4. (K) Avian *Salmonella* S5. (L) Avian *Salmonella* S8. (M) Avian *Salmonella* S10. (N) Avian *Salmonella* S11. (O) Avian *Salmonella* S18. (P) Avian *Salmonella* S19. (Q) Avian *Salmonella* S20. (R) Avian *Salmonella* S22. (S) Avian *Salmonella* S42. (T) Avian *Salmonella* S44. (U) Avian *Salmonella* S104. (V) Avian *Salmonella* S105. (W) Avian *Salmonella* S224. (X) Avian *Salmonella* S278. (Y) Avian *Salmonella* S359. (Z) Avian *Salmonella* S527. Note, strain information is provided in Table 1.
