# Supplementary material for: Characterization and genomic analysis of Salmonella Abortusequi phage, vB_SalP_LDDK01, and its biocontrol application in donkey meat
Source: Front Cell Infect Microbiol. 2024 Dec 23;14:1527201. doi: 10.3389/fcimb.2024.1527201 (PMC11700823; doi:10.3389/fcimb.2024.1527201)
Supplement: Supplementary file 2 [file Table1.docx]

**Supplementary Table S1. Functional annotation of the phage LDDK01 genome**

| **ORF** | **Strand** | **Initiation site** | **Termination site** | **Length/amino acid** | **Annotation Feature** | **Scientific Name** | **Query Cover** | **E value** | **Per. Ident** | **Accession** |
| --- | --- | --- | --- | --- | --- | --- | --- | --- | --- | --- |
| 1 | － | 47 | 196 | 49 | hypothetical protein | *Salmonella* phage vB_SenS_4FS1 | 100.00% | 2E-70 | 100.00% | PP537413.1 |
| 2 | ＋ | 195 | 866 | 223 | putative DNA-binding protein | *Salmonella* phage vB_SenS_4FS1 | 100.00% | 0 | 100.00% | [PP537413.1](https://www.ncbi.nlm.nih.gov/nucleotide/PP537413.1?report=genbank&log$=nucltop&blast_rank=2&RID=EW414V0S013) |
| 3 | － | 901 | 2070 | 389 | putative tail protein | *Salmonella* phage vB_SenS_4FS1 | 100.00% | 0 | 100.00% | [PP537413.1](https://www.ncbi.nlm.nih.gov/nucleotide/PP537413.1?report=genbank&log$=nucltop&blast_rank=2&RID=EW42R5TR013) |
| 4 | － | 2070 | 2489 | 139 | phage tail terminator-like protein | *Salmonella* phage blauehaus | 100.00% | 0 | 100.00% | [NC_073173.1](https://www.ncbi.nlm.nih.gov/nucleotide/NC_073173.1?report=genbank&log$=nucltop&blast_rank=1&RID=EW4AVBVE016) |
| 5 | － | 2489 | 2884 | 131 | hypothetical protein | *Salmonella* phage BPS11T2 | 100.00% | 0 | 100.00% | [MG646668.1](https://www.ncbi.nlm.nih.gov/nucleotide/MG646668.1?report=genbank&log$=nucltop&blast_rank=2&RID=EW56EPC9016) |
| 6 | － | 2881 | 3240 | 119 | tail completion or Neck1 protein | *Salmonella* phage vB_SenS_4FS1 | 100.00% | 0 | 100.00% | [PP537413.1](https://www.ncbi.nlm.nih.gov/nucleotide/PP537413.1?report=genbank&log$=nucltop&blast_rank=2&RID=EW57X67V013) |
| 7 | － | 3240 | 3845 | 201 | hypothetical protein | *Salmonella* phage vB_SenS_4FS1 | 100.00% | 0 | 100.00% | [PP537413.1](https://www.ncbi.nlm.nih.gov/nucleotide/PP537413.1?report=genbank&log$=nucltop&blast_rank=2&RID=EW5DVN5X013) |
| 8 | － | 3848 | 4354 | 168 | hypothetical protein | *Salmonella* phage vB_SenS_4FS1 | 100.00% | 0 | 100.00% | [PP537413.1](https://www.ncbi.nlm.nih.gov/nucleotide/PP537413.1?report=genbank&log$=nucltop&blast_rank=2&RID=EW6GRHGE013) |
| 9 | － | 4497 | 4691 | 64 | hypothetical protein | *Salmonella* phage vB_SenS_4FS1 | 100.00% | 7E-95 | 100.00% | [PP537413.1](https://www.ncbi.nlm.nih.gov/nucleotide/PP537413.1?report=genbank&log$=nucltop&blast_rank=2&RID=EW6K3D69013) |
| 10 | － | 4728 | 5078 | 116 | hypothetical protein | *Salmonella* phage vB_SenS_4FS1 | 100.00% | 0 | 100.00% | [PP537413.1](https://www.ncbi.nlm.nih.gov/nucleotide/PP537413.1?report=genbank&log$=nucltop&blast_rank=2&RID=EW6MYZG2013) |
| 11 | － | 5090 | 5374 | 94 | hypothetical protein | *Salmonella* phage vB_SenS-EnJE1 | 100.00% | 1E-144 | 100.00% | [NC_073187.1](https://www.ncbi.nlm.nih.gov/nucleotide/NC_073187.1?report=genbank&log$=nucltop&blast_rank=1&RID=EW6ZHSSV013) |
| 12 | － | 5435 | 6484 | 349 | major capsid protein | *Salmonella* phage vB_SenS_4FS1 | 100.00% | 0 | 100.00% | [PP537413.1](https://www.ncbi.nlm.nih.gov/nucleotide/PP537413.1?report=genbank&log$=nucltop&blast_rank=2&RID=EW71BR72013) |
| 13 | － | 6488 | 7189 | 233 | hypothetical protein | *Salmonella* phage vB_SpuP_Spp11 | 100.00% | 0 | 100.00% | [MN722429.1](https://www.ncbi.nlm.nih.gov/nucleotide/MN722429.1?report=genbank&log$=nucltop&blast_rank=1&RID=EW792Z73013) |
| 14 | － | 7383 | 7769 | 128 | hypothetical protein | *Salmonella* phage vB_SenS_4FS1 | 100.00% | 0 | 100.00% | [PP537413.1](https://www.ncbi.nlm.nih.gov/nucleotide/PP537413.1?report=genbank&log$=nucltop&blast_rank=2&RID=EW7M2CPC013) |
| 15 | － | 8088 | 8546 | 152 | neck whiskers protein | *Salmonella* phage vB_SenS_4FS1 | 100.00% | 0 | 100.00% | [PP537413.1](https://www.ncbi.nlm.nih.gov/nucleotide/PP537413.1?report=genbank&log$=nucltop&blast_rank=2&RID=EW7XNE3U013) |
| 16 | － | 8549 | 9622 | 357 | head morphogenesis protein | *Salmonella* phage vB_SenS_4FS1 | 100.00% | 0 | 100.00% | [PP537413.1](https://www.ncbi.nlm.nih.gov/nucleotide/PP537413.1?report=genbank&log$=nucltop&blast_rank=2&RID=EW7ZB22Z016) |
| 17 | ＋ | 9731 | 10408 | 225 | amidase | Select seq PP537413.1 | 100.00% | 0 | 100.00% | [PP537413.1](https://www.ncbi.nlm.nih.gov/nucleotide/PP537413.1?report=genbank&log$=nucltop&blast_rank=2&RID=EWR9EN8F013) |
| 18 | － | 10439 | 11911 | 490 | DUF4055 domain-containing protein | *Salmonella* phage vB_SenS_4FS1 | 100.00% | 0 | 100.00% | [PP537413.1](https://www.ncbi.nlm.nih.gov/nucleotide/PP537413.1?report=genbank&log$=nucltop&blast_rank=2&RID=EW861R9E013) |
| 19 | － | 11924 | 13195 | 423 | terminase large subunit | *Salmonella* phage vB_SenS_4FS1 | 100.00% | 0 | 100.00% | [PP537413.1](https://www.ncbi.nlm.nih.gov/nucleotide/PP537413.1?report=genbank&log$=nucltop&blast_rank=2&RID=EW8XBZ6K016) |
| 20 | － | 13185 | 13691 | 168 | hypothetical protein | *Salmonella* phage vB_SenS_4FS1 | 100.00% | 0 | 100.00% | [PP537413.1](https://www.ncbi.nlm.nih.gov/nucleotide/PP537413.1?report=genbank&log$=nucltop&blast_rank=2&RID=EW95CERD013) |
| 21 | － | 13824 | 14054 | 76 | hypothetical protein | Select seq PP537413.1 | 100.00% | 8E-115 | 100.00% | [PP537413.1](https://www.ncbi.nlm.nih.gov/nucleotide/PP537413.1?report=genbank&log$=nucltop&blast_rank=2&RID=EWRM5E9V013) |
| 22 | － | 14068 | 14367 | 99 | NinH protein | *Salmonella* phage vB_SenS_4FS1 | 100.00% | 5E-153 | 100.00% | [PP537413.1](https://www.ncbi.nlm.nih.gov/nucleotide/PP537413.1?report=genbank&log$=nucltop&blast_rank=2&RID=EW98XSAG016) |
| 23 | － | 14367 | 14591 | 74 | NinH family protein | *Salmonella* phage vB_SenS-EnJE6 | 100.00% | 2E-111 | 100.00% | [NC_073191.1](https://www.ncbi.nlm.nih.gov/nucleotide/NC_073191.1?report=genbank&log$=nucltop&blast_rank=1&RID=EW9PS45Z013) |
| 24 | － | 14734 | 14913 | 59 | putative NinZ-like protein | Select seq PP537413.1 | 100.00% | 1E-86 | 100.00% | [PP537413.1](https://www.ncbi.nlm.nih.gov/nucleotide/PP537413.1?report=genbank&log$=nucltop&blast_rank=2&RID=EWS2G40H013) |
| 25 | － | 14910 | 15065 | 51 | DUF2737 family protein | *Salmonella* phage vB_SenS_4FS1 | 100.00% | 2E-73 | 100.00% | [PP537413.1](https://www.ncbi.nlm.nih.gov/nucleotide/PP537413.1?report=genbank&log$=nucltop&blast_rank=2&RID=EW9UMDA9013) |
| 26 | － | 15062 | 15247 | 61 | hypothetical protein | *Salmonella* phage vB_SenS_4FS1 | 100.00% | 6E-90 | 100.00% | [PP537413.1](https://www.ncbi.nlm.nih.gov/nucleotide/PP537413.1?report=genbank&log$=nucltop&blast_rank=2&RID=EWA2AWNH013) |
| 27 | － | 15432 | 15920 | 162 | hypothetical protein | *Salmonella* phage vB_SenS_4FS1 | 100.00% | 0 | 99.59% | [PP537413.1](https://www.ncbi.nlm.nih.gov/nucleotide/PP537413.1?report=genbank&log$=nucltop&blast_rank=2&RID=EWAC997F013) |
| 28 | － | 15898 | 16188 | 96 | putative class I holin | *Salmonella* phage vB_SenS_4FS1 | 100.00% | 5E-148 | 100.00% | [PP537413.1](https://www.ncbi.nlm.nih.gov/nucleotide/PP537413.1?report=genbank&log$=nucltop&blast_rank=2&RID=EWAEMV4B016) |
| 29 | － | 16190 | 16471 | 93 | lysin1 | *Salmonella* phage vB_SenS_4FS1 | 100.00% | 2E-141 | 99.65% | [PP537413.1](https://www.ncbi.nlm.nih.gov/nucleotide/PP537413.1?report=genbank&log$=nucltop&blast_rank=2&RID=EWAG71RA013) |
| 30 | － | 16550 | 16915 | 121 | hypothetical protein | *Salmonella* phage vB_SenS_4FS1 | 100.00% | 0 | 100.00% | [PP537413.1](https://www.ncbi.nlm.nih.gov/nucleotide/PP537413.1?report=genbank&log$=nucltop&blast_rank=2&RID=EWAJ5Y0W013) |
| 31 | － | 16918 | 17121 | 67 | hypothetical protein | *Salmonella* phage vB_SenS_4FS1 | 100.00% | 7E-100 | 100.00% | [PP537413.1](https://www.ncbi.nlm.nih.gov/nucleotide/PP537413.1?report=genbank&log$=nucltop&blast_rank=2&RID=EWCJ3251016) |
| 32 | － | 17118 | 17456 | 112 | hypothetical protein | *Salmonella* phage vB_SenS_4FS1 | 100.00% | 1E-174 | 100.00% | [PP537413.1](https://www.ncbi.nlm.nih.gov/nucleotide/PP537413.1?report=genbank&log$=nucltop&blast_rank=2&RID=EWCJ74ZB016) |
| 33 | － | 17626 | 17790 | 54 | hypothetical protein | *Salmonella* phage vB_SenS_4FS1 | 100.00% | 3E-78 | 100.00% | [PP537413.1](https://www.ncbi.nlm.nih.gov/nucleotide/PP537413.1?report=genbank&log$=nucltop&blast_rank=2&RID=EWCKXTG0016) |
| 34 | ＋ | 18769 | 18954 | 61 | hypothetical protein | *Salmonella* phage vB_SenS_4FS1 | 100.00% | 6E-90 | 100.00% | [PP537413.1](https://www.ncbi.nlm.nih.gov/nucleotide/PP537413.1?report=genbank&log$=nucltop&blast_rank=2&RID=EWCNM7HZ013) |
| 35 | ＋ | 18951 | 19184 | 77 | putative UvsX-like protein | *Salmonella* phage vB_SenS_4FS1 | 100.00% | 2E-116 | 100.00% | [PP537413.1](https://www.ncbi.nlm.nih.gov/nucleotide/PP537413.1?report=genbank&log$=nucltop&blast_rank=2&RID=EWCRDS42016) |
| 36 | ＋ | 19241 | 21427 | 728 | primase/helicase | *Salmonella* phage vB_SenS_4FS1 | 100.00% | 0 | 100.00% | [PP537413.1](https://www.ncbi.nlm.nih.gov/nucleotide/PP537413.1?report=genbank&log$=nucltop&blast_rank=2&RID=EWDAST0P016) |
| 37 | － | 21442 | 21660 | 72 | helix-turn-helix transcriptional regulator | *Salmonella* phage vB_SpuP_Spp11 | 100.00% | 3E-108 | 100.00% | MN722429.1 |
| 38 | ＋ | 21794 | 22306 | 170 | hypothetical protein | *Salmonella* phage vB_SenS_4FS1 | 100.00% | 0 | 100.00% | [PP537413.1](https://www.ncbi.nlm.nih.gov/nucleotide/PP537413.1?report=genbank&log$=nucltop&blast_rank=2&RID=EWDCG6S6016) |
| 39 | ＋ | 22348 | 23622 | 424 | hypothetical protein | *Salmonella* phage PIZ SAE-01E2 | 64.00% | 0 | 99.76% | [MN336266.1](https://www.ncbi.nlm.nih.gov/nucleotide/MN336266.1?report=genbank&log$=nucltop&blast_rank=2&RID=EWDEY4PD016) |
| 40 | ＋ | 23704 | 24330 | 208 | DUF2815 family protein | *Salmonella* phage PJNS016 | 100.00% | 0 | 100.00% | [PP723078.1](https://www.ncbi.nlm.nih.gov/nucleotide/PP723078.1?report=genbank&log$=nucltop&blast_rank=2&RID=EWDJG5DT013) |
| 41 | ＋ | 24388 | 27486 | 1032 | intein-containing DNA polymerase precursor | *Salmonella* phage vB_SenS_4FS1 | 100.00% | 0 | 98.74% | [PP537413.1](https://www.ncbi.nlm.nih.gov/nucleotide/PP537413.1?report=genbank&log$=nucltop&blast_rank=2&RID=EWE9ACK2013) |
| 42 | ＋ | 27573 | 27860 | 95 | putative restriction endonuclease | *Salmonella* phage vB_SenS-EnJE6 | 100.00% | 2E-146 | 100.00% | [NC_073191.1](https://www.ncbi.nlm.nih.gov/nucleotide/NC_073191.1?report=genbank&log$=nucltop&blast_rank=1&RID=EWEB37P6016) |
| 43 | ＋ | 27892 | 28083 | 63 | hypothetical protein | *Salmonella* phage vB_SenS_4FS1 | 100.00% | 3E-93 | 100.00% | [PP932687.1](https://www.ncbi.nlm.nih.gov/nucleotide/PP932687.1?report=genbank&log$=nucltop&blast_rank=1&RID=EWPU0SVJ013) |
| 44 | ＋ | 28085 | 28582 | 165 | hypothetical protein | *Salmonella* phage vB_SenS-EnJE1 | 100.00% | 0 | 100.00% | [NC_073187.1](https://www.ncbi.nlm.nih.gov/nucleotide/NC_073187.1?report=genbank&log$=nucltop&blast_rank=1&RID=EWPU443Z016) |
| 45 | ＋ | 28579 | 31044 | 821 | hypothetical protein | *Salmonella* phage vB_SenS_4FS1 | 100.00% | 0 | 99.96% | [PP537413.1](https://www.ncbi.nlm.nih.gov/nucleotide/PP537413.1?report=genbank&log$=nucltop&blast_rank=2&RID=EWPPDJ8A016) |
| 46 | ＋ | 31041 | 31262 | 73 | hypothetical protein | *Salmonella* phage wast | 100.00% | 7E-110 | 100.00% | [NC_073174.1](https://www.ncbi.nlm.nih.gov/nucleotide/NC_073174.1?report=genbank&log$=nucltop&blast_rank=1&RID=EWMFM9Y9013) |
| 47 | － | 31381 | 33411 | 676 | endorhamnosidase | *Salmonella* phage vB_SenS_4FS1 | 100.00% | 0 | 99.90% | [PP537413.1](https://www.ncbi.nlm.nih.gov/nucleotide/PP537413.1?report=genbank&log$=nucltop&blast_rank=2&RID=EWM94XAF013) |
| 48 | － | 33448 | 35934 | 828 | hypothetical protein | *Salmonella* phage vB_SenS_4FS1 | 100.00% | 0 | 99.12% | [PP537413.1](https://www.ncbi.nlm.nih.gov/nucleotide/PP537413.1?report=genbank&log$=nucltop&blast_rank=2&RID=EWK589KB013) |
| 49 | － | 35997 | 36362 | 121 | hypothetical protein | *Salmonella* phage vB_SenS_4FS1 | 100.00% | 0 | 100.00% | [PP537413.1](https://www.ncbi.nlm.nih.gov/nucleotide/PP537413.1?report=genbank&log$=nucltop&blast_rank=2&RID=EWJVG28Z013) |
| 50 | － | 36359 | 36874 | 171 | minor tail protein | *Salmonella* phage vB_SenS_4FS1 | 100.00% | 0 | 100.00% | [PP537413.1](https://www.ncbi.nlm.nih.gov/nucleotide/PP537413.1?report=genbank&log$=nucltop&blast_rank=2&RID=EWJNFZJU013) |
| 51 | － | 36871 | 37371 | 166 | hypothetical protein | *Salmonella* phage vB_SenS_4FS1 | 100.00% | 0 | 100.00% | PP537413.1 |
| 52 | － | 37373 | 39706 | 777 | tape measure protein | *Salmonella* phage vB_SenS_4FS1 | 100.00% | 0 | 100.00% | [PP537413.1](https://www.ncbi.nlm.nih.gov/nucleotide/PP537413.1?report=genbank&log$=nucltop&blast_rank=2&RID=EWHCK28V013) |
| 53 | － | 39699 | 40058 | 119 | hypothetical protein | *Salmonella* phage vB_SenS_4FS1 | 100.00% | 0 | 100.00% | [PP537413.1](https://www.ncbi.nlm.nih.gov/nucleotide/PP537413.1?report=genbank&log$=nucltop&blast_rank=2&RID=EWHAT0J8013) |
| 54 | － | 40064 | 40480 | 138 | tail assembly chaperone | *Salmonella* phage vB_SenS_4FS1 | 100.00% | 0 | 100.00% | [PP537413.1](https://www.ncbi.nlm.nih.gov/nucleotide/PP537413.1?report=genbank&log$=nucltop&blast_rank=2&RID=EWG893WG016) |
| 55 | ＋ | 40650 | 40829 | 59 | superinfection immunity protein | *Salmonella* phage skrot | 100.00% | 1E-86 | 100.00% | [NC_073172.1](https://www.ncbi.nlm.nih.gov/nucleotide/NC_073172.1?report=genbank&log$=nucltop&blast_rank=1&RID=EWG58246016) |
| 56 | ＋ | 40892 | 42046 | 384 | calcineurin-like phosphoesterase superfamily domain protein | *Salmonella* phage vB_SpuP_Spp11 | 100.00% | 0 | 100.00% | [MN722429.1](https://www.ncbi.nlm.nih.gov/nucleotide/MN722429.1?report=genbank&log$=nucltop&blast_rank=1&RID=EWR99JVJ013) |
| 57 | ＋ | 42116 | 42346 | 76 | hypothetical protein | *Salmonella* phage vB_SpuP_Spp11 | 100.00% | 8E-115 | 100.00% | [MN722429.1](https://www.ncbi.nlm.nih.gov/nucleotide/MN722429.1?report=genbank&log$=nucltop&blast_rank=1&RID=EWG35P02016) |
